# What does not kill it makes it weaker: effects of sub-lethal concentrations of ivermectin on the locomotor activity of *Anopheles aquasalis*

**DOI:** 10.1186/s13071-017-2563-0

**Published:** 2017-12-28

**Authors:** Vanderson de Souza Sampaio, Gustavo Bueno da Silva Rivas, Kevin Kobylinski, Yudi Tatiana Pinilla, Paulo Filemon Paolluci Pimenta, José Bento Pereira Lima, Rafaela Vieira Bruno, Marcus Vinícius Guimarães Lacerda, Wuelton Marcelo Monteiro

**Affiliations:** 1 Departamento de Ensino e Pesquisa, Fundação de Medicina Tropical Dr. Heitor Vieira Dourado, Manaus, Brazil; 2 Escola de Ciências da Saúde, Universidade do Estado do Amazonas, Manaus, Brazil; 3 Sala de Análise de Situação em Saúde, Fundação de Vigilância em Saúde do Amazonas, Manaus, Brazil; 4 Department of Entomology and Nematology, Citrus Research and Education Center, University of Florida, Lake Alfred, FL USA; 5 Armed Forces Research Institute of Medical Sciences, Bangkok, Thailand; 6 Centro de Pesquisa René Rachou, Fundação Oswaldo Cruz (Fiocruz), Belo Horizonte, Brazil; 7 Instituto Oswaldo Cruz, Fundação Oswaldo Cruz (Fiocruz), Rio de Janeiro, Brazil; 8 Instituto de Pesquisa Leônidas & Maria Deane, Fundação Oswaldo Cruz (Fiocruz), Manaus, Brazil

**Keywords:** Malaria elimination, Vector control, Ivermectin, *Anopheles aquasalis*, Locomotor activity, Amazon

## Abstract

**Background:**

Malaria remains a major public health concern. Vector control measures based solely on insecticide treated nets (ITNs) and indoor residual spraying (IRS) have demonstrated not to be feasible for malaria elimination. It has been shown that ivermectin affects several aspects of *Anopheles* species biology. Along the Latin American seacoast, *Anopheles aquasalis* Curry plays an important role in malaria transmission. The observation of mosquitoes locomotor activity under laboratory conditions can reveal details of their daily activity rhythms, which is controlled by an endogenous circadian clock that seems to be influenced by external signals, such as light and temperature. In this study, we assessed basal locomotor activity and the effects of ivermectin on locomotor activity of the American malaria vector, *An. aquasalis*.

**Methods:**

Adult females of *Anopheles aquasalis* used in experiments were three to five days post-emergence. Blood from one single subject was used to provide mosquito meals by membrane feeding assays. Powdered ivermectin compound was used to achieve different concentrations of drug as previously described. Fully engorged mosquitoes were individually placed into glass tubes and provided with 10% sucrose. Each tube was placed into a Locomotor Activity Monitor (LAM). The LAMs were kept inside an incubator under a constant temperature and a 12:12 h light:dark cycle. The average locomotor activity was calculated as the mean number of movements performed per mosquito in the period considered. Intervals of time assessed were adapted from a previous study. One-way ANOVA tests were performed in order to compare means between groups. Additionally, Dunnett’s method was used for *post-hoc* pairwise means comparisons between each group and control. Stata software version 13 was used for the analysis.

**Results:**

*Anopheles aquasalis* showed a nocturnal and bimodal pattern for mosquitoes fed both control blood meals and sub-lethal concentrations of ivermectin. In this species, activity peaks occurred at the beginning of the photophase and scotophase in the control group. The nocturnal activity is evident and higher just after the evening peak and maintains basal levels of locomotion throughout the scotophase. In the entire group analysis, locomotor activity means of experimental sets were significantly lower than control for each period of time evaluated. In the survival group, the locomotor activity means of all treatment sets were lower than control mosquitoes for all intervals of time when both the whole period and scotophase were assessed. When the middle of scotophase was evaluated, means were significantly lower for LC_15_ and LC_25_, but not LC_5_. For the beginning of photophase period, significant differences were detected only between control and LC_5_. When both the photophase and scotophase were assessed alone, no significant differences were found. Mean locomotor activity was significantly lower for dead group when compared to survival group for all experimental sets when whole period, photophase, and scotophase were assessed.

**Conclusions:**

Ivermectin seems to decrease locomotor activity of *An. aquasalis* at sub-lethal concentrations. The effects on locomotor activity increase according at higher ivermectin concentrations and are most evident during the whole scotophase as well as in the beginning and in the end of this phase, and sub-lethal effects may still be observed in the photophase. Findings presented in this study demonstrate that sub-lethal ivermectin effects reduce mosquito locomotor activity, which could diminish vectorial capacity and therefore the malaria transmission.

**Electronic supplementary material:**

The online version of this article (10.1186/s13071-017-2563-0) contains supplementary material, which is available to authorized users.

## Background

The World Health Organization (WHO) estimated that 214 million cases of malaria occurred worldwide in 2015, considering it a major public health concern [1]. Two major goals appear to be on both research centers and policy makers agenda worldwide: (i) reducing malaria burden to elimination levels in high-incidence countries, and (ii) eliminating malaria in those where transmission levels are already low. Once lowered transmission levels are achieved, malaria eradication strategies should be implemented [2–5].

Malaria elimination will require interventions that are able to overcome residual transmission, reducing the reservoir of infection, the time that a person or a mosquito is infectious, and the rate at which infections are spread. This goal can be achieved by drugs or vaccines directed against the parasite or by new tools that attack the vector, combined with improved diagnostics and surveillance [6, 7]. Mass drug administration (MDA) with antimalarial drugs, mass screening and treatment (MSAT), focused screening and treatment (FSAT) and reactive case detection (RCD) are based on rapid diagnostic and timely treatment. Although reported as having no effects when applied as stand-alone strategies, modelling suggests they could be effective when used concomitantly with vector control tools [8]. Traditional vector control measures, insecticide treated nets (ITNs) and indoor residual spraying (IRS), may not achieve malaria elimination by themselves. Extensive use of ITNs and IRS have promoted changes in vector behavior from indoor to outdoor feeding and resting [9, 10], requiring novel vector control interventions which can specifically target outdoor feeding and resting vectors.

It has been shown that ivermectin affects several aspects of *Anopheles* species biology that are critical for malaria transmission, including the daily probability of adult mosquito survivorship, daily probability that a mosquito feeds on a human host, vector competence, and vector density in relation to the host [11–18]. Since ivermectin is administered to mosquitoes through the host blood, acting as a systemic insecticide, it would affect both indoor and outdoor transmission regardless of when host seeking occurs [11]. Ivermectin has also been shown to be effective against a range of diseases, including onchocerciasis [19]. The Onchocerciasis Elimination Program for the Americas (OEPA) in the Brazilian Amazon endemic area has proven to be effective and safe in indigenous Yanomami communities where two rounds of ivermectin MDA are administered per year [20, 21]. Taking that into account, ivermectin MDA could be a promising strategy to be implemented in Latin America for malaria elimination.

It has been demonstrated that ivermectin MDA to humans in West Africa decreases adult mosquito survival rates, reduces the proportion of older females, thereby shifting the population age structure which reduces the sporozoite rate [12, 16, 22]. Furthermore, ivermectin MDA can target vectors which prefer to feed or rest outdoors. Such features are in agreement with Malaria Eradication Research Agenda (malERA) initiative recommendations as well as WHO preferred product characteristics guideline on endectocides for malaria control [4, 23]. Ivermectin MDA in conjunction with ACT MDA was predicted via modelling to decrease malaria transmission as well as significantly reduce the time and minimal number of MDA rounds necessary to achieve elimination [8].

In this study, we assessed ivermectin effect on the locomotor activity of the American malaria vector *Anopheles aquasalis* Curry. *Anopheles aquasalis* plays an important role in malaria transmission in coastal regions of Latin America, from Central America to southern Brazil [24–26]. The zoophagic tendencies of *An. aquasalis* [27] make treatment of livestock with endectocides a particularly attractive vector control option. Ivermectin has been demonstrated to decrease *An. aquasalis* survivorship when blood-fed on treated volunteer blood from 4 h to 14 days post-ingestion. Sub lethal effects of ivermectin on several aspects of malaria vectors such as fecundity, knockdown, delay in recovery, delay in re-feeding, sporontocidal effect, among others, have been shown. These findings show that even at low concentrations the drug can potentially influence malaria transmission [11, 28–30]. Locomotor activity is an important aspect of vectorial capacity that is largely influenced by the circadian clock. Consequently, factors that influence this endogenous pacemaker can have a direct impact on malaria transmission [31, 32]. Despite being well studied in many species [33], no evidences regarding the effects of ivermectin on locomotor activity are available and this study aims to assess effects of sublethal concentrations of the drug on this important indicator.

## Methods

### Mosquito colony

*Anopheles aquasalis* specimens were obtained from a colony at the Laboratory of Physiology and Control of Arthropod Vectors at the Oswaldo Cruz Institute (Fiocruz), Rio de Janeiro, Brazil. Mosquitoes were raised at 26–27 °C, 70–80% relative humidity and 12:12 h light/dark period (starting 8:00 am). Larvae were fed commercial fish food (Tetramin Gold®^,^ Blacksburg, VA, USA) and adults were provided 10% sucrose solution ad libitum. Adult females used in experiments were three to five days post-emergence. Insecticide resistance profile of the colony for pyrethroids and organophosphates showed a high susceptibility for both larvae and adults [34].

### Blood meals

Blood from one single subject (male, 37 years old, 77 kg, 1.82 m height, and body mass index of 23.2) was used to provide mosquito meals by membrane feeding assays.

Powdered ivermectin compound was used to achieve different concentrations of drug as previously described [29]. Sub-lethal concentrations of Ivermectin were assessed at 5-day-lethal concentrations that kill 5% (LC_5_ = 18.28 ng/ml), 15% (LC_15_ = 25.92 ng/ml) and 25% (LC_25_ = 31.92 ng/ml). Control mosquitoes were fed a mixture of blood and dimethyl-sulfoxide diluted in phosphate buffered saline matched to the highest drug concentration (LC_25_). Membrane feeding assays (MFA) were used to feed groups of mosquitoes with blood meals kept at 36 °C throughout the assay. Approximately 50 fully engorged mosquitoes were gently transferred to 500 ml cardboard containers after 30 min and then kept under the same conditions of colonized specimens for 24 h before the locomotor activity assay.

### Locomotor activity assay

Fully engorged females were individually placed into glass tubes (70 × 10 mm) with cotton soaked with 10% sucrose solution sealing one end of the tube. Each tube was placed into a Locomotor Activity Monitor (LAM) with specific silicon trays with infrared light and detectors surrounding them. The infrared beam allows a computer program to count each time a mosquito passes through the beam, individually measuring their locomotor activity [35]. The LAMs were kept inside a Precision Scientific Incubator Mod. 818 under a constant temperature of 25 °C and a photoperiod of 12 h of light followed by a scotoperiod of 12 h of dark (12:12 LD), as described by [36]. Groups of 32 mosquitoes were placed into each LAM in duplicate and activity measured over 96 h. All assays were performed in three replicates.

### Data analysis

The overall locomotor activity for every mosquito was obtained from 30 min intervals (48 data points throughout each 24 h period) in all assays. The average locomotor activity was calculated as the mean number of movements performed per mosquito in the period considered. Adapting the indices used in Lima-Camara et al. [36], the following intervals of time were assessed: (i) overall activity average (mean activity in the whole period); (ii) average activity in photophase (mean activity exclusively with lights on); (iii) average activity in scotophase (mean activity exclusively with lights off); (iv) average activity in the beginning of scotophase (mean activity in the first 30 min of lights off); (v) average activity in the middle of scotophase (mean activity with the lights off except for the first and last 30 min); and (vi) average activity at the beginning of photophase (mean activity in the first 30 min of lights on). For the construction of line graphs of average locomotor activity throughout the period, only mosquitoes that survived until the last day were counted. Mean comparisons were performed considering all mosquitoes assessed (entire group) and mosquitoes that survived until the last day (survival group). Additionally, differences between average locomotor activity from the survival group and only mosquitoes that died between 0 and 4 days of the assay, excluding all mosquitoes that survived (dead group) for all lethal concentrations (LC_5_, LC_15_ and LC_25_) were assessed.

Data normality was assessed by Shapiro-Wilk normality test. One-way ANOVA tests were performed in order to compare means between groups. Additionally, Dunnett’s method was used for *post-hoc* pairwise means comparisons between each group and control. Stata software version 13 (StataCorp. LP, College Station, TX, USA) was used for the analysis.

## Results

**Fig. 1:**
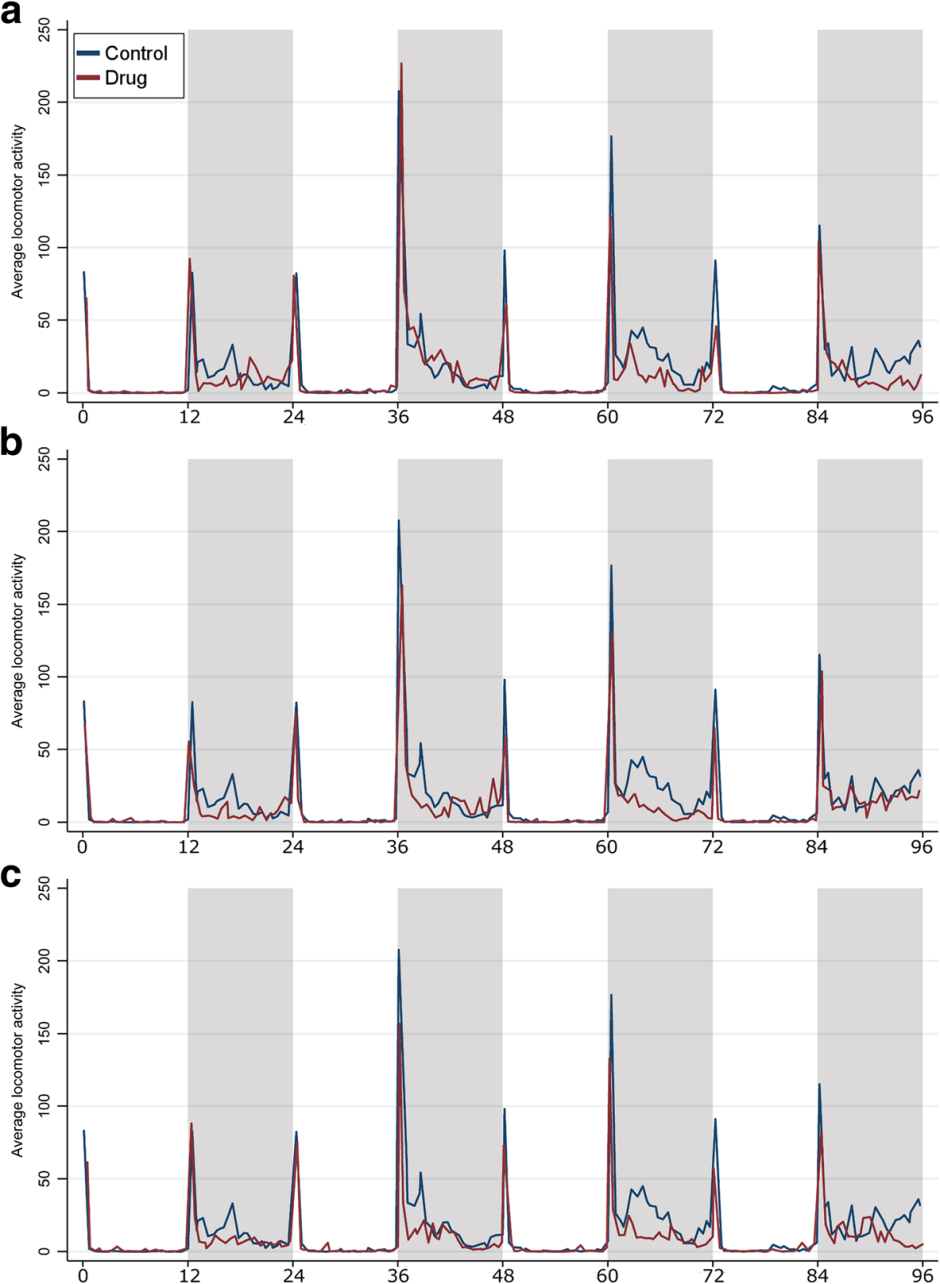
Average locomotor activity throughout the period assessed (day 1 from day 4) of control compared to mosquitoes fed with blood containing ivermectin in: **a** lethal concentration 5 (LC_5_); **b** lethal concentration 15 (LC_15_); and **c** lethal concentration 25 (LC_25_). Light areas on represent photophase and shaded areas the scotophase

*Anopheles aquasalis* showed a nocturnal and bimodal pattern both for control and mosquitoes fed sub-lethal ivermectin concentrations. Activity occurred as major peaks at the beginning of both the scotophase and photophase and a secondary pronounced activity at middle of the scotophase. Intense activity was observed during the whole scotophase, decreasing throughout the dawn. Conversely, mosquitoes had a predominant resting behavior at whole photophase. (Fig. 1). For Control, LC_5_, LC_15_ and LC_25_, the number of mosquitoes was: 56, 64, 48, and 64 in the entire group, respectively, and 40, 37, 34 and 42 for the survival group, respectively (Table 1).

**Table 1:** Locomotor activity means comparisons between groups in different periods

		Whole period	Photophase	Scotophase	Beginning of scotophase	Middle of scotophase	Beginning of photophase
	*n*	Mean ± SE (*P-*value)	Mean ± SE (*P*-value)	Mean ± SE (*P-*value)	Mean ± SE (*P*-value)	Mean ± SE (*P-*value)	Mean ± SE (*P*-value)
Entire group							
Control	56	11.75 ± 1.27 (−)	4.71 ± 0.64 (−)	18.80 ± 2.32 (−)	119.77 ± 14.53 (−)	14.40 ± 2.11 (−)	87.56 ± 11.51 (−)
LC5	64	7.70 ± 0.82 (0.006)	2.90 ± 0.29 (0.006)	12.49 (± 1.58) (0.026)	88.12 ± 11.20 (0.161)	9.01 ± 1.44 (0.04)	51.66 ± 5.52 (0.004)
LC15	48	7.41 ± 0.91 (0.007)	2.86 ± 0.32 (0.009)	11.96 (± 1.64) (0.024)	83.09 ± 11.64 (0.119)	8.79 ± 1.48 (0.04)	52.37 ± 6.61 (0.01)
LC25	64	6.84 ± 0.73 (0.001)	2.88 ± 0.34 (0.005)	10.80 (± 1.30) (0.003)	88.27 ± 11.48 (0.164)	7.53 ± 1.08 (0.005)	51.77 ± 7.15 (0.004)
Survival group							
Control	40	14.18 ± 1.48 (−)	4.69 ± 0.63 (−)	23.66 ± 2.74 (−)	145.58 ± 16.07 (−)	18.34 ± 2.65 (−)	88.65 ± 11.89 (−)
LC5	37	10.73 ± 1.11 (0.03)	3.35 ± 0.40 (0.122)	18.12 ± 2.21 (0.05)	136 ± 14.63 (0.939)	12.88 ± 2.22 (0.165)	63.27 ± 7.28 (0.04)
LC15	34	9.63 ± 1.05 (0.02)	3.42 ± 0.37 (0.163)	15.84 ± 1.93 (0.03)	113.21 ± 13.18 (0.294)	11.52 ± 1.89 (0.05)	66.66 ± 7.24 (0.07)
LC25	42	8.92 ± 0.86 (0.003)	3.67 ± 0.43 (0.282)	14.17 ± 1.62 (0.005)	114.98 ± 14.10 (0.295)	9.89 ± 1.44 (0.01)	66.76 ± 9.24 (0.07)

**Fig. 2:**
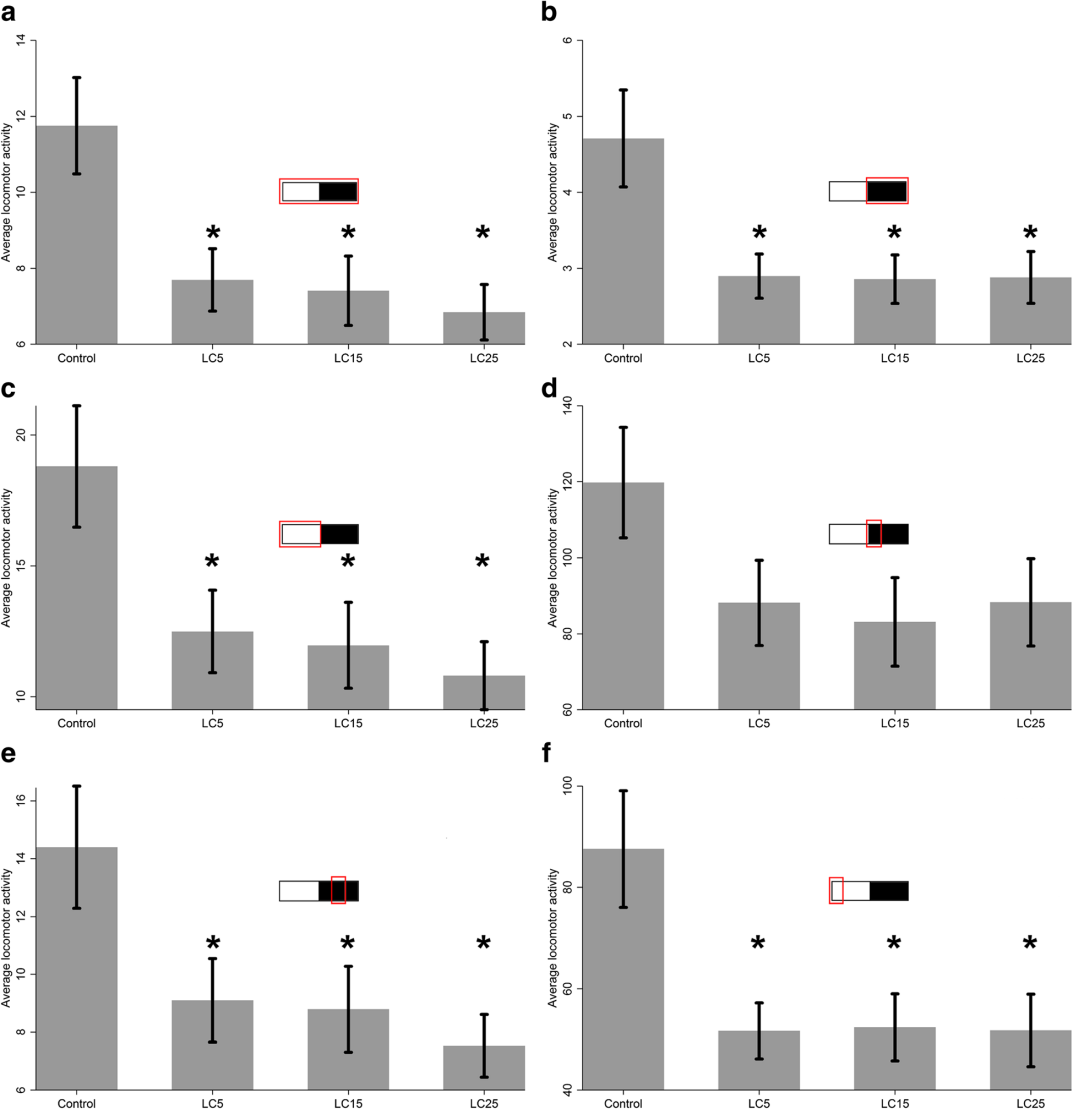
Bar graph comparing mean locomotor activity between control (*n* = 56) and mosquitoes fed with different ivermectin concentration considering all mosquitoes assessed (entire group): LC_5_ (*n* = 64); LC_15_ (*n* = 48); LC_25_ (*n* = 64). Mean comparison of experimental sets at: **a** whole period; **b** scotophase; **c** photophase; **d** beginning of scotophase; **e** middle of scotophase; **f** beginning of photophase. *P < 0.05

**Fig. 3:**
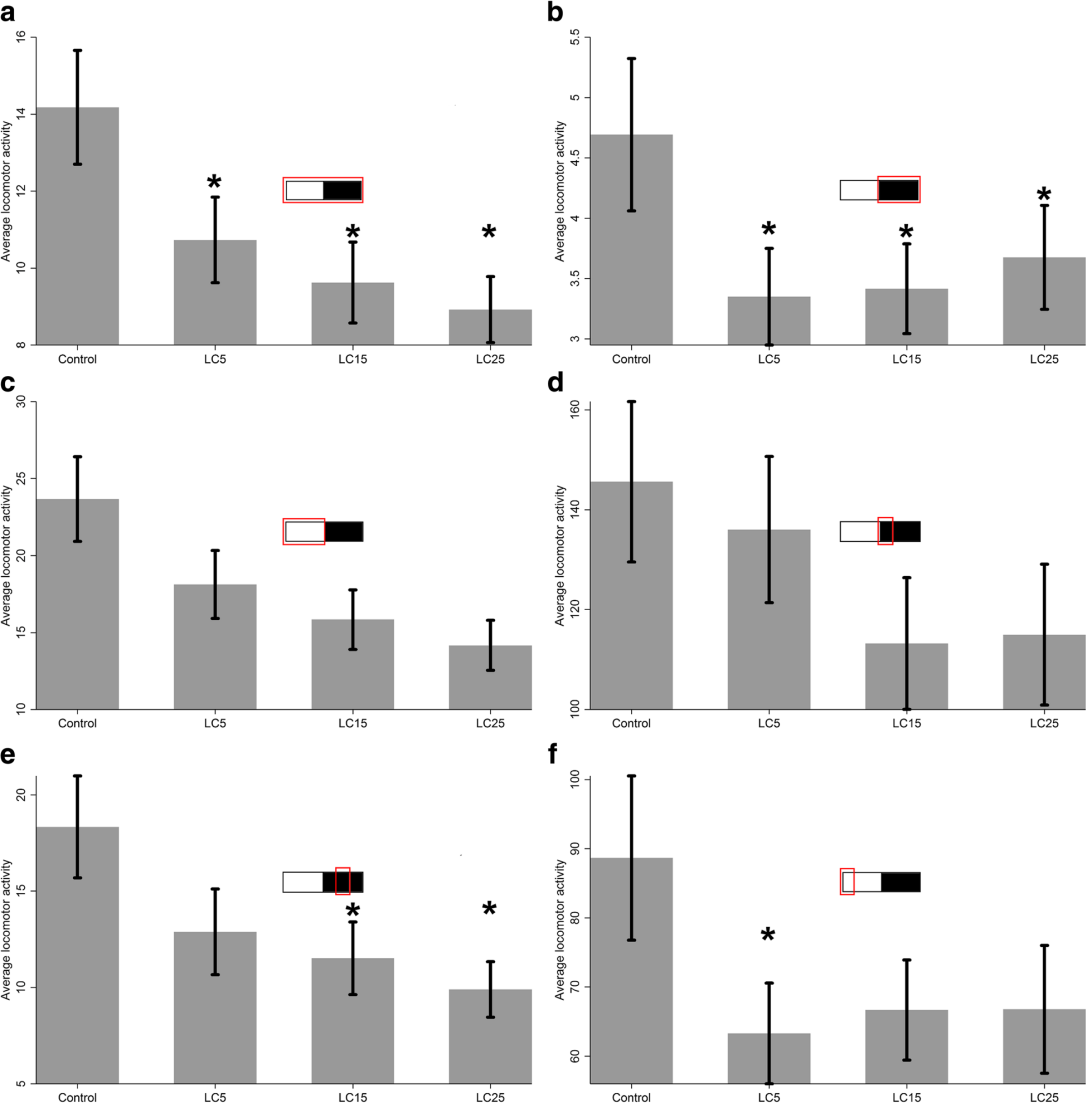
Bar graph comparing mean locomotor activity between control (*n* = 40) groups fed with different ivermectin concentration considering only mosquitoes alive until the last day of observation (survival group): LC_5_ (*n* = 37); LC_15_ (*n* = 34); LC_25_ (*n* = 42). Mean comparison of groups at: **a** whole period; **b** scotophase; **c** photophase; **d** beginning of scotophase; **e** middle of scotophase; **f** beginning of photophase. *P < 0.05

In both the survival group and entire group analyses, the highest levels of activity were observed in the beginning of scotophase and photophase (Table 1, Figs. 2, 3).

In the entire group analysis, locomotor activity means of experimental sets were significantly lower than control for each period of time evaluated (*F*_(3,228)_ = 5.6, *P* < 0.001), except for the beginning of scotophase (Table 1, Fig. 2).

In the survival group, the locomotor activity means of all treatment sets were lower than control mosquitoes for all intervals of time (*F*_(3,149)_ = 4.27, *P* < 0.01) when both the whole period and scotophase were assessed (Table 1, Fig. 3). When the middle of scotophase was evaluated, means were significantly lower for LC_15_ (9.63 ± 1.05) and LC_25_ (8.92 ± 0.86) (*F*_(3,149)_ = 3.8, *P* < 0.01), but not LC_5_ (10.73 ± 1.11). For the beginning of photophase period, significant differences were detected only between control (88.65 ± 11.89) and LC_5_ (63.27 ± 7.28) (*F*_(3,149)_ = 3.2, *P* < 0.01). When the photophase was assessed alone, no significant differences were found (*F*_(3,149)_ = 1.71, *P* > 0.05) (Table 1).

Mean locomotor activity was significantly lower for the dead group when compared to the survival group for all experimental sets when (i) whole period [LC_5_ (3.53 ± 0.63, *P* < 0.001); LC_15_ (2.02 ± 0.60, *P* < 0.001); LC_25_ (2.87 ± 0.88, *P* < 0.001)]; and control (5.69 ± 6.98), *P* < 0.001) (*t*_(62)_ = -5.11, *P* < 0.0001); (ii) photophase [LC_15_ (1.50 ± 0.47, *P* < 0.001); LC_25_ (1.36 ± 0.40, *P* < 0.001)]; and control (4.75 ± 6.52, *P* < 0.001) (*t*_(62)_ = -1.85, *P* < 0.05); and (iii) scotophase [LC_5_ (3.53 ± 0.63, *P* < 0.001); LC_15_ (2.54 ± 0.90, *P* < 0.001); LC_25_ (4.38 ± 1.44, *P* < 0.001)]; and control (6.63 ± 10.01, *P* < 0.001) (*t*_(62)_ = -4.87, *P* < 0.0001) were assessed (Additional file 1: Table S1).

## Discussion

The effectiveness of malaria vector control tools is heavily influenced by mosquito behavior [19]. Although *Anopheles aquasalis* has been reported as an important malaria vector, little is known on its locomotor activity [37]. In *Anopheles gambiae*, the daily flight activity displays a nocturnal and bimodal pattern [38–41]. Peaks in the beginning of both scotophase and photophase, as well as intense activity throughout the scotophase, decreasing over time, has been shown here for *An. aquasalis*, which agrees with studies in other *Anopheles* spp. that showed the same patterns of resting and action, even applying rudimentary methods available at the time. Although the use of laboratory mosquitoes in an artificial environment may be considered a weakness of the study, important information on locomotor activity was provided and may be validated by field studies in the future [38, 42, 43].

Effects of ivermectin on different aspects of vectorial capacity were previously demonstrated [11, 14], including effects on survivorship and reproductive fitness of *Anopheles aquasalis* [29]. The role of ivermectin as a potential transmission blocking tool in *Anopheles aquasalis* infection by *Plasmodium vivax* was demonstrated [44]. Although advances have been made on the knowledge of the ivermectin effects on different aspects of malaria transmission, gaps in the knowledge of its effects on mosquito behavior exist [45].

It was demonstrated here that ivermectin promotes a decline in the overall *Anopheles* locomotor activity peaks during the photophase, the beginning of scotophase as well as throughout the scotophase. Ivermectin not only reduces the morning peak activity, but also the mean activity during the photophase (Table 1, Fig. 1). These results suggest that ivermectin may increase light avoidance in *Anopheles aquasalis*. Jones et al. [46] suggested that light-on peak could be a startle response mediated by the nervous system, while the latency of light-on response and light avoidance would be a hormone mediated process. Thus, considering results presented here, it is plausible to think that ivermectin has influence on both mechanisms [46].

The nocturnal activity of *Anopheles* can be divided in two components: the evening peak and a secondary activity in the middle or late part of the night [38, 39]. Jones & Gubbins [39] reported that inseminated anophelines had a reduction of evening peak, while the secondary nocturnal activity was increased. These findings suggest that the evening peak activity is important for mating, which is reduced in already inseminated females, but the activity in the middle of the night is crucial for blood-feeding. Locomotor activity results presented here show that ivermectin reduces both the evening peak and nocturnal activity. Consequently, it is tempting to suggest that ivermectin reduced locomotor activity could diminish both mating and blood-feeding efficiency.

Effects of sub-lethal ivermectin concentrations on locomotor activity in other species of insects have been demonstrated previously. Ivermectin was shown to inhibit the locomotor activity of *Scarabaeus cicatricosus*, a key dung beetle species in Mediterranean ecosystems, by reducing spontaneous muscle force [47]. A dose-response effect can be observed when different concentrations are compared to each other, demonstrating that the decrease in mosquito activity is due to the drug.

Both for entire group and survival group analyses, control showed higher means when compared to mosquitoes fed ivermectin in almost all the periods evaluated, although not significant for all periods (Table 1). This finding can be explained since the ivermectin mechanism of action involves the activation of receptors for glutamate-gated chloride (GluCl), glycine (Gly), γ-aminobutyric acid (GABA) channels, and modulation of Cys-loop ion channel, affecting both neuronal activity and muscular contractility [48–50]. Additionally, expression of GluCI receptors have been demonstrated in *An. gambiae* thoracic ganglia, an organ evolved in the control of flight and leg muscles. These findings suggest that the disruption of GluCl channels in this organ could lead to muscle paralysis and thus decreased locomotor activity [51]. Although significant differences had been found in most periods for both analyses, it should be emphasized that entire group take into account both mosquitoes that lived and those that died, possibly skewing this result, which may explain the significant differences found between the approaches.

Mean mosquito locomotor activity within each treatment (control, LC_5_, LC_15_, or LC_25_) was compared between the dead group (only mosquitoes dead in the assay) and survival group (only mosquitoes that survived). In all experimental sets there was significantly reduced locomotor activity between the dead group and survival group mosquitoes (except control at photophase), which suggests that any mosquito near death will display reduced locomotor activity regardless of whether they ingested ivermectin (Additional file 1: Table S1).

Reduced locomotor activity caused by ivermectin would likely impact mosquito host seeking, dispersal, and even mating, since mosquitoes under drug effect would be less likely to reach mating swarms. Since the egg laying pattern of *An. aquasalis* was shown to occur predominantly at night [52] and sub-lethal ivermectin reduced locomotor activity during the scotophase, ivermectin would further impact reproductive fitness beyond reduction in fecundity alone observed in Sampaio et al. [29]. A reduction in mosquito egg laying potential and fecundity would reduce mosquito density in relation to humans, which directly impacts vectorial capacity. Furthermore, a reduction in host seeking by ivermectin would delay time for a mosquito to re-feed, which directly impacts vectorial capacity.

Although the time evaluated in this study is not compatible with time to complete the malaria extrinsic incubation period, it seems appropriate to think that the effects demonstrated here should last while ivermectin is present in the mosquito. Additionally, since ivermectin effects on postsynaptic potentials by reducing muscle membrane resistance were shown to be irreversible, it may be that such effects are chronic to the arthropod [53].

## Conclusions

Ivermectin decreases locomotor activity of *Anopheles aquasalis* at sub-lethal concentrations. The effect increases with the concentration and is most evident during the scotophase, especially at the beginning and end of this period, although its effects may still be observed in the photophase. Findings presented in this study show evidence that the effects of ivermectin go far beyond those already presented and affect *Anopheles* locomotion, which could further impact vectorial capacity, reducing malaria transmission. Results presented here reinforce the importance of ivermectin as an oral insecticide and its use in human MDA or mass livestock treatment strategies as a possible complementary tool for malaria elimination. Since *Anopheles* outdoor host-seeking behavior has a major contribution to malaria transmission in Latin America, ivermectin MDA or mass livestock treatment could be very effective. Further studies of this nature, assessing such effects in other *Anopheles* species, must be performed in order to allow estimation of the impact of this on malaria transmission.

## Additional file

Additional file 1: Table S1. Means comparisons between only mosquitoes that died between 0 and 4 days of the assay, excluding all mosquitoes that survived (dead group) and mosquitoes that survived until the last day (survival group) at LC_5_, LC_15_ and LC_25_ lethal concentrations for the whole period, photophase and scotophase. (DOCX 14 kb)

## Abbreviations

ACT: Artemisinin-based combination therapy
ELISA: Enzyme-linked immunosorbent assay
FMT-HVD: Fundação de Medicina Tropical Dr. Heitor Vieira Dourado
FSAT: Focused screening and treatment
LC: Lethal concentration
malERA: Malaria Eradication Research Agenda
MDA: Mass drug administration
MFA: Membrane feeding assay
MSAT: Mass screening and treatment
RCD: Reactive case detection
WHO: World Health Organization
