# Supplementary material for: What does not kill it makes it weaker: effects of sub-lethal concentrations of ivermectin on the locomotor activity of Anopheles aquasalis
Source: Parasit Vectors. 2017 Dec 28;10:623. doi: 10.1186/s13071-017-2563-0 (PMC5745606; doi:10.1186/s13071-017-2563-0)
Supplement: Additional file 1: Table S1. — Means comparisons between only mosquitoes that died between 0 and 4 days of the assay, excluding all mosquitoes that survived (dead group) and mosquitoes that survived until the last day (survival group) at LC5, LC15 and LC25 lethal concentrations for the whole period, photophase and scotophase. (DOCX 14 kb) [file 13071_2017_2563_MOESM1_ESM.docx]

Additional file 1: Table S1. Locomotor activity means comparison between dead group and survival group at LC_5_, LC_15_, and LC_25_

| Whole period | | | | | | Photophase | | | | | Scotophase | | | | | |
| --- | --- | --- | --- | --- | --- | --- | --- | --- | --- | --- | --- | --- | --- | --- | --- | --- |
|  | Dead group^a^ | | Survival group^b^ | |  | Dead group^a^ | | Survival group^b^ | |  | Dead group^a^ | | Survival group^b^ | |  |  |
|  | Mean (SE) | *n* | Mean (SE) | *n* | *P*-value | Mean (SE) | *n* | Mean (SE) | *n* | *P*-value | Mean (SE) | *n* | Mean (SE) | *n* | *P*-value |  |
| Control | 5.69 (± 6.98) | 16 | 14.18 (± 1.48) | 40 | 0.000 | 4.75 (± 6.52) | 16 | 4.69 (± 0.63) | 40 | 0.515 | 6.63 (± 10.01) | 16 | 23.66 (± 2.74) | 40 | 0.000 |  |
| LC_5_ | 3.53 (± 0.63) | 27 | 10.73 (± 1.11) | 37 | 0.000 | 2.28 (± 0.39) | 27 | 3.35 (± 0.40) | 37 | 0.030 | 3.53 (± 0.63) | 27 | 18.12 (± 2.21) | 37 | 0.000 |  |
| LC_15_ | 2.02 (± 0.60) | 14 | 9.63 (± 1.05) | 34 | 0.000 | 1.50 (± 0.47) | 14 | 3.42 (± 0.37) | 34 | 0.002 | 2.54 (± 0.90) | 14 | 15.84 (± 1.93) | 34 | 0.000 |  |
| LC_25_ | 2.87 (± 0.88) | 22 | 8.92 (± 0.86) | 42 | 0.000 | 1.36 (± 0.40) | 22 | 3.68 (± 0.43) | 42 | 0.000 | 4.38 (± 1.44) | 22 | 14.17 (± 1.62) | 42 | 0.000 |  |

^a^Dead group: Only mosquitoes that died during the assay, excluding all mosquitoes that survived

^b^Survival group: mosquitoes that survived until the last day of experiment

*Abbreviation*: SE, standard error
